# Improved sensing characteristics of dual-gate transistor sensor using silicon nanowire arrays defined by nanoimprint lithography

**DOI:** 10.1080/14686996.2016.1253409

**Published:** 2017-01-06

**Authors:** Cheol-Min Lim, In-Kyu Lee, Ki Joong Lee, Young Kyoung Oh, Yong-Beom Shin, Won-Ju Cho

**Affiliations:** ^a^ Department of Electronic Materials Engineering, Kwangwoon University, Seoul, Republic of Korea; ^b^ Hazards Monitoring BioNano Research Center, Korea Research Institute of Bioscience & Biotechnology (KRIBB), Daejeon, Republic of Korea

**Keywords:** Dual-gate field-effect transistor, silicon nanowire, nanoimprint lithography, ion-sensitive field-effect transistor, pH sensor, capacitive coupling, 40 Optical, magnetic and electronic device materials, 201 Electronics, Semiconductor / TCOs, 600 Others: electronic device

## Abstract

This work describes the construction of a sensitive, stable, and label-free sensor based on a dual-gate field-effect transistor (DG FET), in which uniformly distributed and size-controlled silicon nanowire (SiNW) arrays by nanoimprint lithography act as conductor channels. Compared to previous DG FETs with a planar-type silicon channel layer, the constructed SiNW DG FETs exhibited superior electrical properties including a higher capacitive-coupling ratio of 18.0 and a lower off-state leakage current under high-temperature stress. In addition, while the conventional planar single-gate (SG) FET- and planar DG FET-based pH sensors showed the sensitivities of 56.7 mV/pH and 439.3 mV/pH, respectively, the SiNW DG FET-based pH sensors showed not only a higher sensitivity of 984.1 mV/pH, but also a lower drift rate of 0.8% for pH-sensitivity. This demonstrates that the SiNW DG FETs simultaneously achieve high sensitivity and stability, with significant potential for future biosensing applications.

## Graphical abstract

**Figure:**
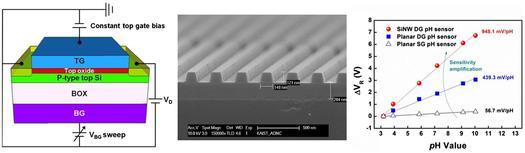

## 1. Introduction

In recent years, the demand for biosensors has increased rapidly because of aging societies. In particular, label-free biosensors based on field-effect transistors (FETs) have attracted considerable attention as potential candidates for point-of-care biosensing applications, having numerous merits such as rapid label-free detection, miniaturized sensor size, and portability.[1–6] During the development of high-quality and reliable FET-based biosensors, sensitivity and stability are among the most important factors under consideration. However, achieving both sensitivity and stability simultaneously is difficult because of the trade-off relationship between these properties.[7] Stability is generally influenced by changes in the sensitivity. When signal amplitude is increased by amplification circuits or by enlarging the sensor surface area, the noise in the system is increased as well, and no enhancement of the signal-to-noise (S/N) ratio occurs.

In order to attain an optimum balance between sensitivity and stability, and thereby increase the S/N ratio, dual-gate FETs (DG FETs) have been developed.[8–10] DG FETs can amplify the signal by several times through a capacitive-coupling effect, which is induced by their unique asymmetric structure between the top and bottom gates.[11] In our previous works, we have already demonstrated that the S/N ratio is ultimately enhanced because DG FETs amplify the signal relatively more largely than the noise.[12–14] However, DG FETs still have some room for further improvement of their performance.

Silicon nanowire-based FETs (SiNW FETs) have recently drawn attention as promising biosensor tools because of their ultrahigh sensitivity, selectivity, and size compatibility.[15–17] SiNW FETs offer an increased capacitance in their nanowire geometry as well as an outstanding charge controllability and a low off-state leakage current due to their high surface-area-to-volume ratios.[18–21] Nevertheless, the availability of SiNW FETs is limited by the difficulty of the manufacturing process for the devices. Fabrication of SiNW FETs follows a bottom-up or top-down approach. SiNW bottom-up growth processes, such as the vapor-liquid-solid (VLS) growth technique, plasma-enhanced chemical vapor deposition (PECVD), and layer-by-layer self-assembly, face difficulties in device integration because the size and positions of the SiNWs cannot always be perfectly controlled. Top-down approaches, such as electron-beam lithography, focused-ion-beam lithography, and deep-UV photolithography, allow SiNW printing to obtain the desired shapes and structures, but are limited by low throughput and high cost because of the serial ‘writing’ processing and the use of expensive instrumentation; hence, such devices are limited to use in research settings.[22]

Nanoimprint lithography (NIL) is a simple nanolithography process that transfers patterns by pressing a designed master mold into the resist; this technique has been proposed to overcome the limited production volume and high cost of other top-down techniques.[23] Compared to conventional nanopatterning techniques, NIL has higher throughput, lower fabrication cost, and excellent reproducibility.[24,25] Moreover, it can be used to fabricate not only uniformly distributed and size-controlled nanowires over a large area but also various high-resolution nanoscale patterns.[26]

Here, we design DG FETs based on SiNWs formed using NIL and compare the performance of these devices with conventional planar DG FETs. By applying the DG FETs in pH sensors, we investigate the potential of SiNW DG FETs in future biological and chemical sensors.

## 2. Experimental section

### 2.1. Formation of SiNW on semiconductor-on-insulator (SOI) wafer

**Figure 1.**
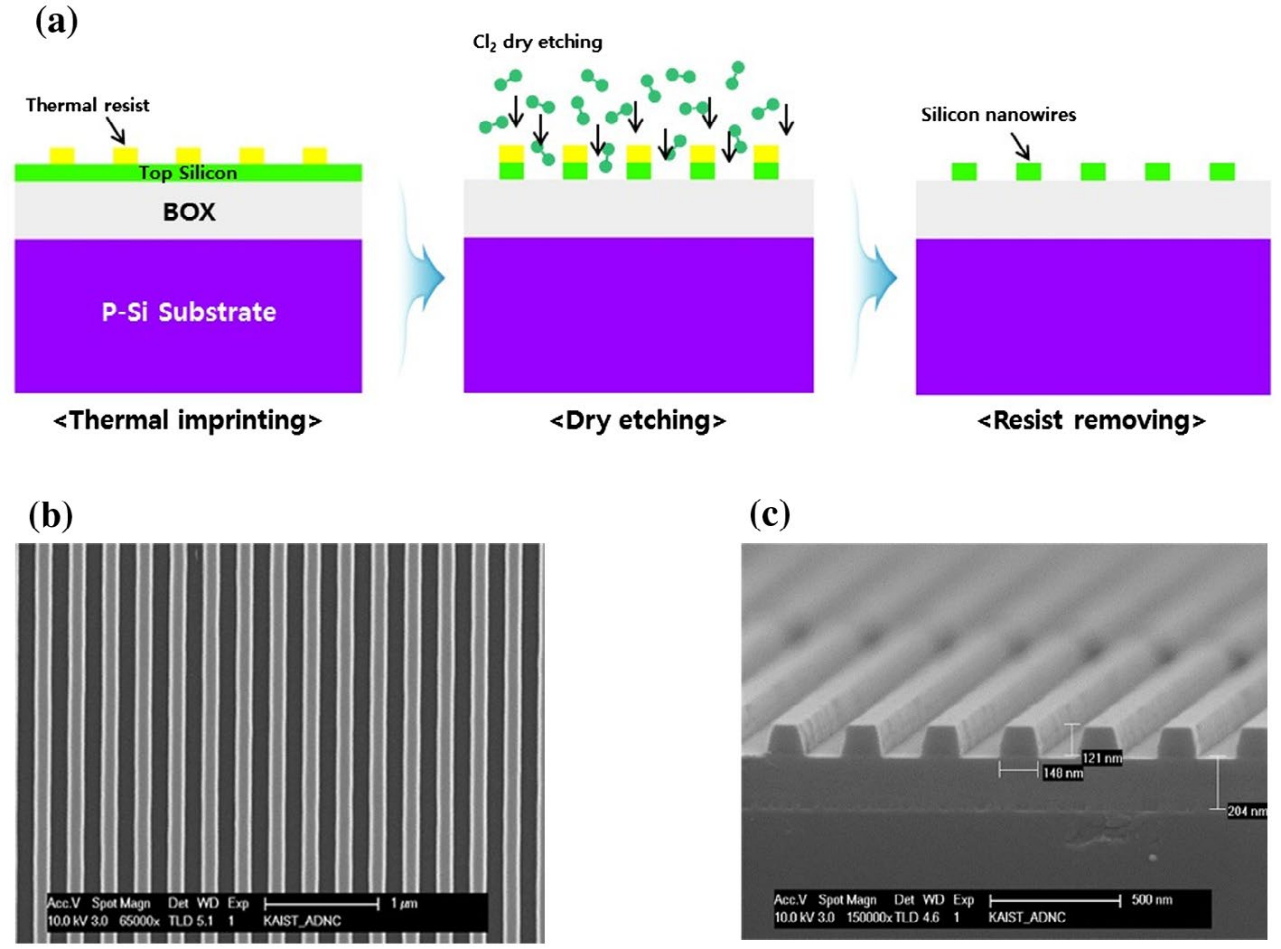
(a) Schematic of the fabrication process of SiNWs by NIL, involving ICP dry-etching. (b) SEM image of nanoimprinted SiNWs. (c) SEM image of the cross-section of the SiNWs fabricated on SOI wafer.

The fabrication process of SiNWs is shown in Figure 1(a). A p-type (100) silicon-on-insulator (SOI) wafer with a 200-nm-thick buried oxide (BOX) layer was used as the base substrate. The thickness of the top silicon layer of the SOI wafer was ~120 nm and the resistivity and doping level were 10 Ω cm and 1 × 10^15^ cm^−3^, respectively. The substrate was cleaned in acetone and isopropanol for 10 min and rinsed with deionized water for 5 min, before drying with nitrogen. To decrease the hydrophobicity of the substrate, it was treated with oxygen plasma (PINK GmbH, Wertheim, Germany, plasma-finish) for 10 s at 300 W, pressure of 80 Pa, and an O_2_ gas flow rate of 300 ml min^–1^. A 200-nm-thick poly(methyl methacrylate) (PMMA) (Micro resist, Berlin, Germany, PMMA35k300) layer was spin-coated onto the glass substrate of the control sample at 3000 rpm for 30 s and baked at 120°C for 120 s using a hot plate. A polycarbonate (PC) film mold was pressed using a nanoimprinter at 4.5 bar for 140 min. The transfer temperature was 130°C, above the glass transition temperature T_g_ of PMMA. The PC film mold and the imprinted polymer were cooled below T_g_ of the polymer to preserve the imprinted pattern after mold release at 90°C. The thermal resin layer formed by the imprinted linear arrays was successfully formed on the substrate. After imprinting, inductively coupled plasma (ICP) dry etching of Si using Cl_2_ (20 sccm)/Ar (40 sccm) plasma (Oxford Plasmalab, Bedford, USA 100) was performed with a pressure of 3 mTorr and source and bias powers of 1000 W (top) and 400 W (bottom), respectively. The remaining PMMA layer was removed by an acetone solution under sonication. Figure 1(b) and 1(c) show scanning electron microscopy (SEM) images of the SiNWs formed by NIL.

### 2.2. Fabrication of the FET-based sensor

FET-based sensors were designed using standard metal-oxide semiconductor field-effect transistors (MOSFETs) and contained a FET and sensing membrane as transducer and sensing regions, respectively.[27] In this work, we separated the sensing membrane from the FET to allow the reusability of the FET, disposability of the sensing membrane, and minimization of ion damage to the FET caused by undesirable ions on the sensing membrane.[28]

#### 2.2.1. Fabrication of SiNW FET (transducer region)

**Figure 2.**
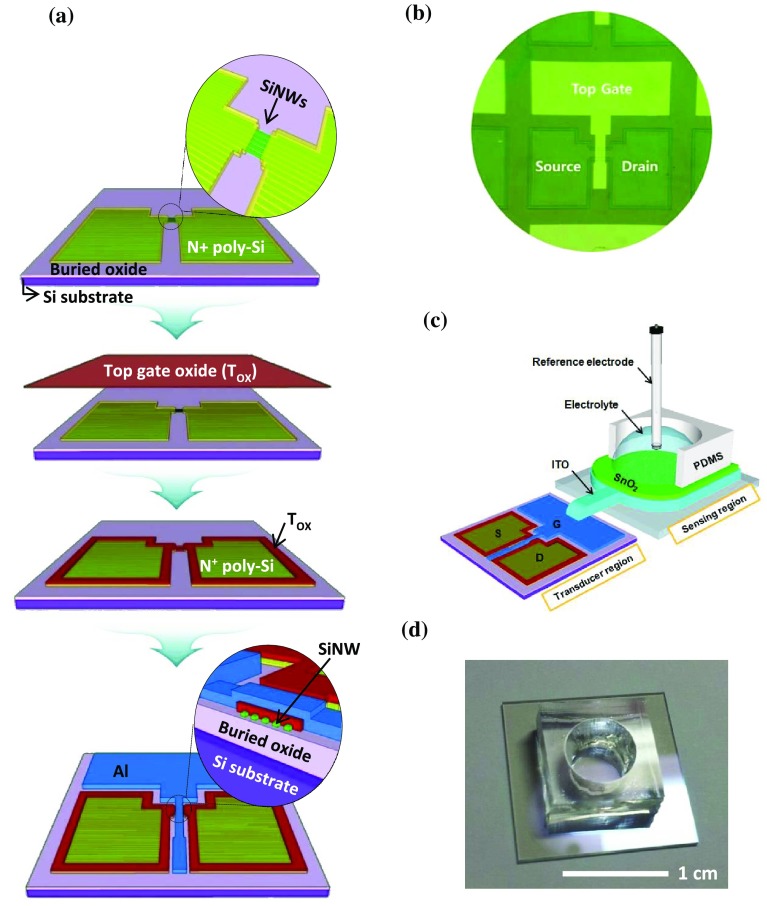
(a) Fabrication process flow diagram for nanoimprinted SiNW FETs. (b) Optical microscope image of the fabricated SiNW FETs. (c) Schematic of SiNW FET-based sensor with disposable sensing region. (d) Photograph of the sensing region of FET-based sensor.

Figure 2(a) shows a flow diagram of the fabrication of SiNW FETs. SiNWs formed by NIL were used for the channel layer of the FET. After forming an active region by photolithography and a reactive ion etching (RIE) process, a 100-nm-thick phosphorus-doped polycrystalline silicon (N^+^ poly-Si) was deposited at the source and drain regions using low-pressure chemical vapor deposition (LPCVD). Next, a 20-nm-thick SiO_2_ layer for the top-gate oxide (T_ox_) was grown by thermal oxidation. To reduce the defect density and improve the electrical properties of the devices, rapid thermal annealing (RTA) was performed at 850°C for 30 s in N_2_/O_2_ ambient gas. A 150 nm-thick aluminum gate electrode was formed using an e-beam evaporator. Finally, forming gas annealing was performed at 400°C for 30 min in 2% H_2_/N_2_ ambient to improve the interfacial quality between the top/bottom gate oxides and channel layer. The channel length and width of the fabricated FET were 10 μm and 20 μm, respectively. An optical microscope image of the fabricated SiNW FETs is shown in Figure 2(b).

#### 2.2.2. Fabrication of the sensing membrane (sensing region)

A 100-nm-thick indium tin oxide (ITO) metal electrode, for transferring surface potential variations on the sensing membrane to the gate electrode of the FET, was deposited on a glass substrate using a radio-frequency (RF) sputter process. Subsequently, a 50-nm-thick tin oxide (SnO_2_) layer was deposited by RF magnetron sputtering at room temperature. SnO_2_ was used as the sensing membrane because previous studies have demonstrated the exceptional long-term stability and reliability of the material.[29,30] During sputtering, the RF power, chamber pressure, and Ar gas flow rate were maintained at 50 W, 3 mTorr, and 20 sccm, respectively. Finally, a polydimethylsiloxane (PDMS) chamber for the injection of the pH solution was attached to the sensing membrane using silicone glue. The inside diameter of the active region in the chamber was 0.6 cm. The ITO electrode in the sensing region was directly connected to the aluminum gate electrode in the transducer region using an electric wire. A schematic of the fabricated SiNW FET-based sensor and a photograph of the sensing region are shown in Figure 2(c) and 2(d), respectively.

### 2.3. Operational mechanism of the DG FET-based sensor

The fabricated FET-based sensors could be operated in both single-gate (SG) and dual-gate (DG) modes (Figure S1). Conventional FET-based sensors are generally driven in SG mode with the maximum achievable sensitivity, or Nernstian sensitivity, limited to 59 mV/pH at 25°C, because sensitivity ($\Delta V_{th}^{T}$) is determined only by changes in the surface potential (Δ*ψ*_0_) of the sensing membrane, which can be described as $\Delta V_{th}^{T}=-\Delta \psi_{0}$.[16,31] The FET-based sensors proposed in this study are driven in DG mode; the sensitivity ($\Delta V_{th}^{B}$) depends not only on but also on the capacitive-coupling ratio (C_top_/C_bottom_), representing the amplification factor of the sensor, between the top and bottom gate capacitances; this can be described as follows:

$$\Delta V_{th}^{B}=-\frac{C_{top}}{C_{bottom}}\Delta \psi_{0}=\frac{C_{top}}{C_{bottom}}\Delta V_{th}^{T}$$

where C_top_ and C_bottom_ denote the top and bottom gate capacitances per unit area, respectively.[13,32] Consequently, the DG FET-based sensor can achieve highly enhanced sensitivity, exceeding the Nernst limit of 59 mV/pH, if C_top_ is much greater than C_bottom_.

### 2.4. Measurement of the FET-based sensor

The drain current versus gate voltage (I_D_-V_G_) curves in the electrical characteristics evaluation and the pH sensing test were measured by an Agilent 4156B semiconductor parameter analyzer. A commercial Ag/AgCl electrode was used as the reference electrode; all measurements were conducted in a dark box to avoid interference by light and external noise.

## 3. Results and discussion

### 3.1. Electrical characteristics of the fabricated FETs

**Figure 3.**
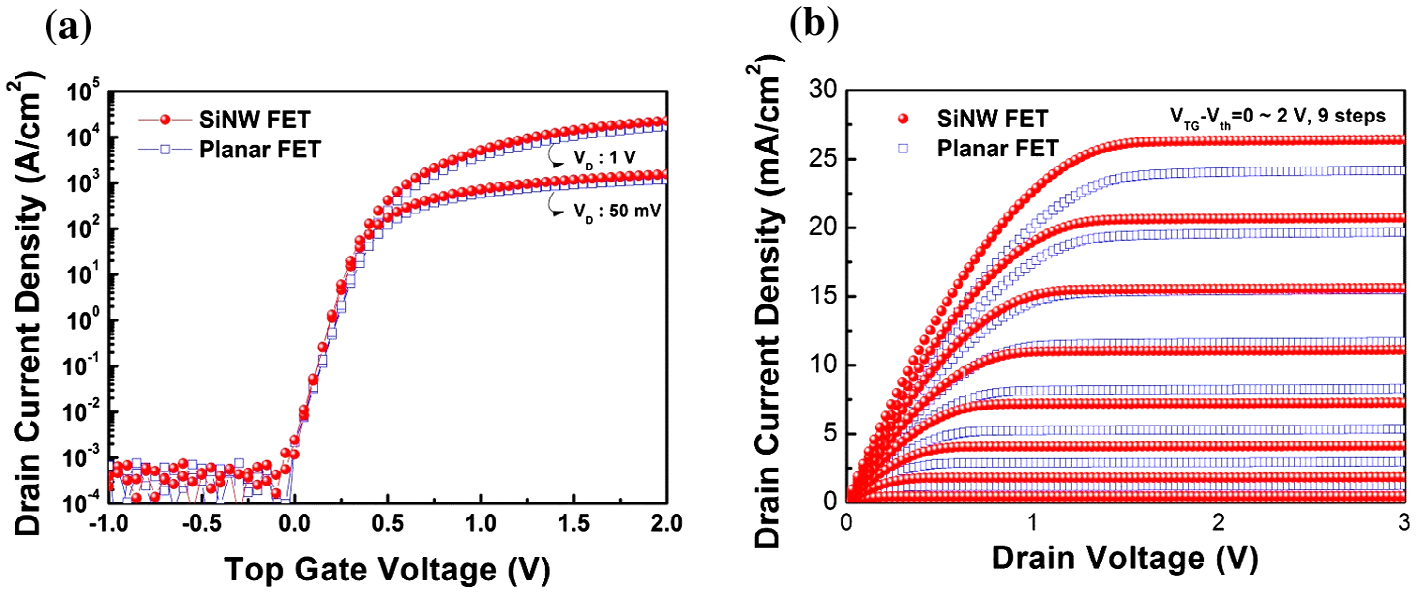
(a) Transfer behavior for constant V_D_ (50 mV and 1 V) of planar FETs and SiNW FETs. (b) Electrical output characterization of planar FETs and SiNW FETs. The gate voltage is varied from 0 to 2 V in steps of 0.25 V.

Figure 3 depicts (a) the transfer behavior (I_D_-V_G_) and (b) the output characteristic (I_D_-V_D_) curves of the fabricated planar and SiNW FETs. As observed from the transfer behavior, both devices have strong gate dependences. In addition, the output characteristics show that the drain current is effectively controlled by several constant gate bias voltages (V_G_ from 0 to 2 V with a step of 0.25 V). The extracted electrical parameters are summarized in Table 1. Compared to the planar FETs, the SiNW FETs exhibit superior electrical characteristics, including a higher field-effect mobility of 730.3 cm^2^ V^–1^ s^–1^, lower threshold voltage (V_th_) of 54.7 mV, steeper subthreshold swing (SS) of 76.0 mV/dec, larger on/off current ratio of 2.3 × 10^8^, and lower interface trap density (D_it_) of 2.8 × 10^12^ cm^−2^ eV^−1^. D_it_ between the top silicon channel and top oxide was calculated by:

$$D_{it}=\frac{SS\times C_{i}\log\left(e\right)}{q\times k_{B}T}$$

**Table 1.** Electrical parameters of planar and SiNW FETs.

	Mobility (cm^2^ V^–1^·s^–1^)	*V*_th_ (mV)	*SS* (mV/dec)	On/off current ratio	*D*_it_ (cm^−2^·eV^−1^)
Planar FET	438.3	94.4	80.0	1.7 × 10^8^	2.9 × 10^12^
SiNW FET	730.3	54.7	76.0	2.3 × 10^8^	2.8 × 10^12^

where C_i_ denotes the capacitance per unit area, q is the electron charge, k_B_ is the Boltzmann’s constant, and T is the absolute temperature.

### 3.2. Signal amplification capabilities of the FETs in DG operation

**Figure 4.**
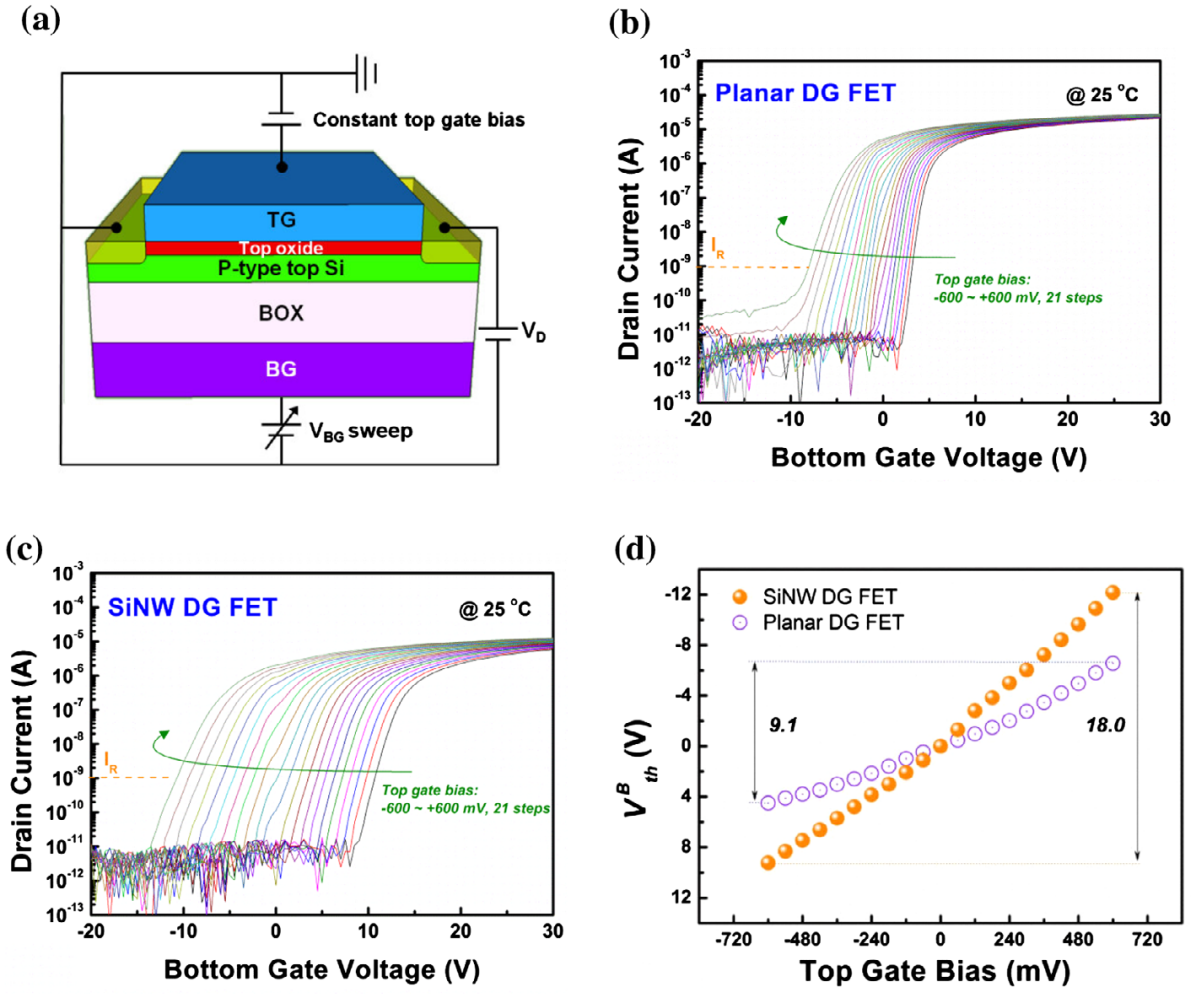
(a) Schematic of FETs in dual-gate (DG) operation. Transfer curves of (b) planar DG FETs and (c) SiNW DG FETs with constant top gate biases from –600 mV to 600 mV in steps of 60 mV, as indicated by the arrow. The drain bias is 50 mV. (d) Top gate bias versus $V_{th}^{B}$ plot for planar and SiNW DG FETs. $V_{th}^{B}$ for each top gate bias is defined as the bottom-gate voltage corresponding to I_D_ of 1 nA.

Figure 4(a) depicts a schematic of planar and SiNW FETs in DG operation (hereafter, referred to as planar or SiNW DG FETs). To compare the signal amplification capabilities of the devices in DG mode, we observed the I_D_-V_G_ curves with varying the top gate bias from –600 mV to 600 mV in steps of 60 mV. Figure 4(b) and 4(c) show the I_D_-V_G_ curves of the planar and SiNW DG FETs as functions of the top gate bias. In both devices, $V_{th}^{B}$ is shifted to the negative direction as the top gate bias is changed from –600 mV to 600 mV. This is because the top surface region of the p-type top silicon layer is depleted by the electrons induced by the positive top-gate bias; near the region, a negative-charged space-charge region is developed that assists with channel formation during bottom-gate sweeping.[10,33,34]

Figure 4(d) shows the variation of $V_{th}^{B}$ for planar and SiNW DG FETs as a function of top-gate bias variation. The ratios of the top-gate bias to $V_{th}^{B}$ variation, or the capacitive-coupling ratios, for the planar and SiNW DG FETs are 9.1 and 18.0 respectively. The SiNW DG FETs have higher capacitive-coupling ratios than planar DG FETs because the former has a larger top surface area and smaller bottom surface area, leading to an increase in C_top_ and a decrease C_bottom_ (Figure S2).

### 3.3. Stability test of the devices under high-temperature stress

**Figure 5.**
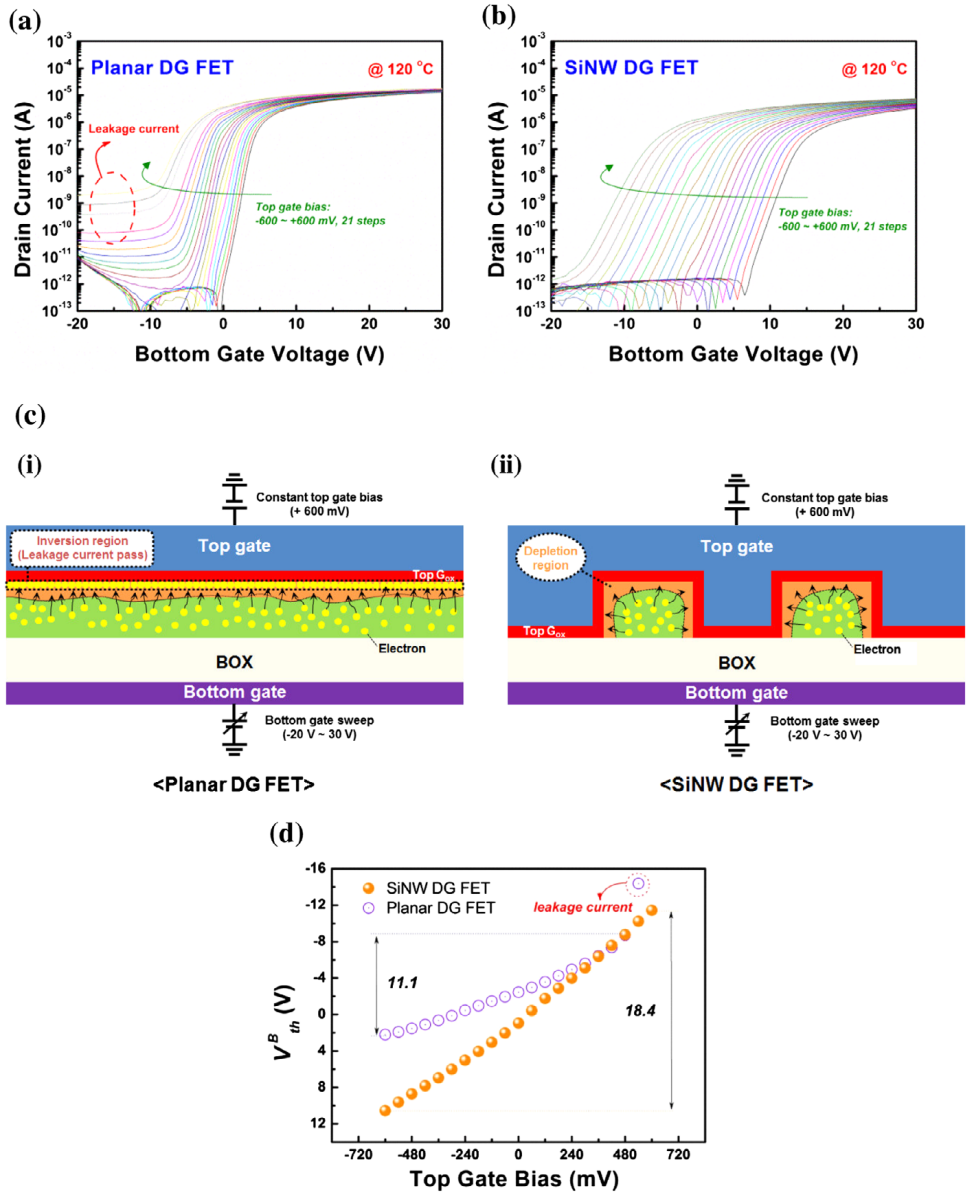
I_D_-V_G_ curves for (a) planar DG FETs and (b) SiNW DG FETs measured at 120°C with constant top gate biases ranging from –600 mV to + 600 mV in steps of + 60 mV as indicated by the arrow. The drain bias is set at 50 mV. (c) Schematic illustration of the operation principle of a planar DG FET and SiNW DG FET measured at 120°C with constant top gate bias of + 600 mV. (d) Top gate bias versus $V_{th}^{B}$ plot of planar DG FETs and SiNW DG FETs measured at 120°C.

In order to evaluate the stability of the planar and SiNW FETs, we observed the electrical performances of the devices under high-temperature stress. Figure 5 shows the I_D_-V_G_ curves of planar and SiNW DG FETs measured at 120°C. Here, an interesting phenomenon is observed in the results: planar DG FETs have large off-state leakage currents with increases in the top gate, but SiNW DG FETs show no corresponding leakage. The off-state leakage current in the planar DG FETs is attributed to the many electrons, generated by the high-temperature stress, forming a surface inversion region that acts as a leakage current path, as shown in Figure 5(c)(i) in the top silicon channel layer. In contrast, in the SiNW DG FETs, no surface inversion region is developed because the energy of the top gate bias is distributed over a wide surface area of SiNWs, as shown in Figure 5(c)(ii) (supplementary data are shown in Figure S3).

Figure 5(d) displays the $\Delta V_{th}^{B}$ variation for planar and SiNW DG FETs as functions of the top gate bias variation measured at 120°C. The capacitive-coupling ratios of the planar and SiNW DG FETs are shown to be 11.1 and 18.4, respectively. Table 2 summarizes the capacitive-coupling ratios of the planar and SiNW DG FETs, measured at room temperature and 120°C. SiNW DG FETs exhibit a small error percentage of 2.6%, whereas planar DG FETs have a relatively larger error of 22.2%. This implies that the SiNW DG FETs have better stability and reliability than the planar DG FETs do.

**Table 2.** Capacitive-coupling ratios of planar and SiNW DG FETs measured at room temperature and 120°C.

	Capacitive-coupling ratio at 25°C	Capacitive-coupling ratio at 120°C	Δ Capacitive-coupling ratio	Error percentage (%)
Planar DG FET	9.1	11.1	2.0	22.2
SiNW DG FET	18.0	18.4	0.5	2.6

### 3.4. pH sensing test

**Figure 6.**
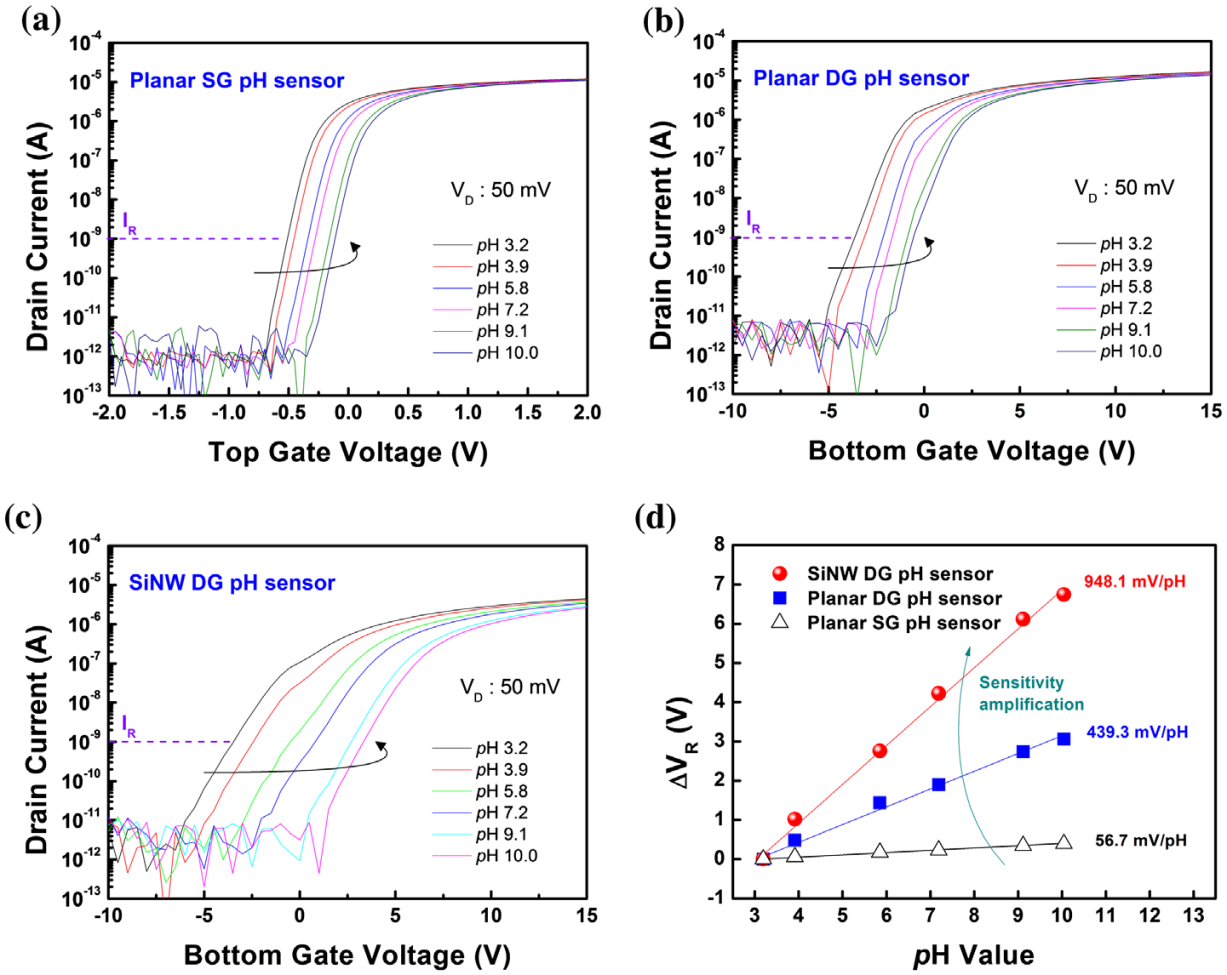
I_D_-V_G_ curves of (a) planar SG pH sensors, (b) planar DG pH sensors, and (c) SiNW DG pH sensors for a large pH range (3–10). All measurements performed at V_D_ = 50 mV and conducted three times to verify the reproducibility. (d) Change in the response voltage (V_R_) of planar and SiNW pH sensors for a wide range of pH (3–10). V_R_ for each pH buffer solution defined as a corresponding gate voltage to reference drain current (I_R_) of 1 nA.

Figure 6 shows the I_D_-V_G_ curves of (a) planar SG FET-based pH sensors (hereafter referred to as planar SG pH sensors), (b) planar DG pH sensors, and (c) SiNW DG pH sensors, measured in solutions with different pH values. As shown, V_th_ is positively shifted for all pH sensors, with shifting behavior based on the variation in pH between 3 and 10. Figure 6(d) shows the change in the response voltage (V_R_) of the planar and SiNW pH sensors based on the pH variation. As shown, while the planar SG pH sensors have a low sensitivity of 56.7 mV/pH within the Nernst limit of 59 mV/pH, the planar DG pH sensors exhibit a high sensitivity of 439.3 mV/pH, far beyond the conventional pH response limit. In particular, the sensitivity (984.1 mV/pH) of the SiNW DG pH sensors is amplified significantly more than that of the conventional planar DG pH sensors, because they have such high capacitive-coupling ratios. The measured sensing parameters are summarized in Table 3 (I_D_-V_G_ curves of SiNW SG pH sensors are shown in Figure S4).

**Table 3.** Sensing parameters of planar and SiNW pH sensors measured in each operation mode.

	Operation mode	pH-sensitivity (mV/pH)	Drift rate (mV h^–1^)	Drift rate for pH-sensitivity (%)
Planar pH sensor	SG	56.7	1.9	3.3
	DG	439.3	8.0	1.8
SiNW pH sensor	SG	56.9	1.8	3.2
	DG	984.1	8.3	0.8

**Figure 7.**
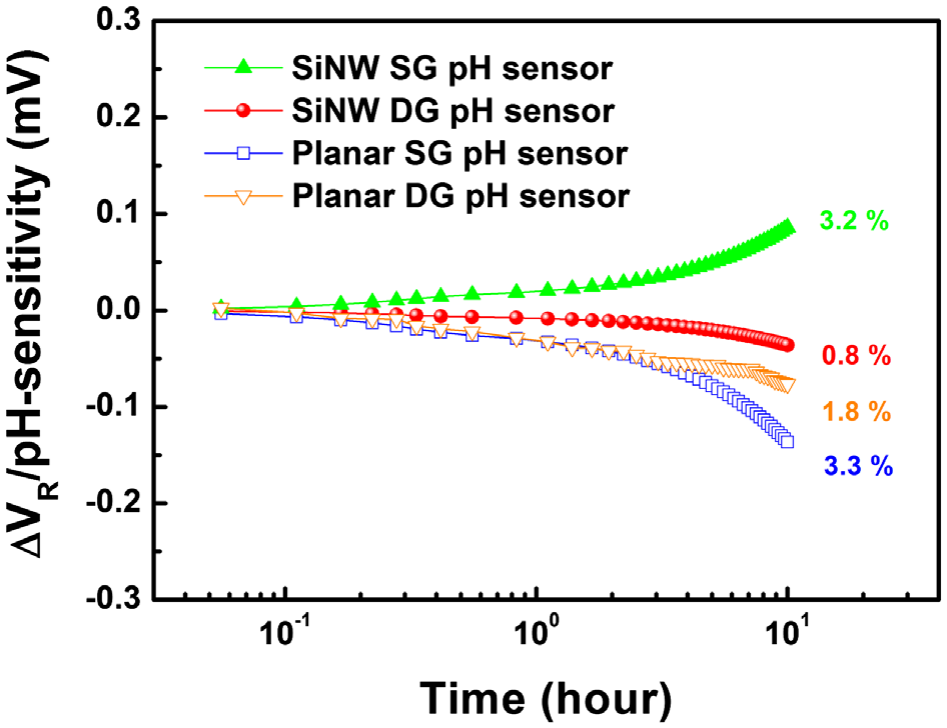
Drift characteristics of planar and SiNW pH sensors in SG and DG modes measured in a pH 7 solution for 10 h.

In order to evaluate the long-term chemical stability of the fabricated pH sensors, we investigated the drift characteristics by observing the shift of V_th_ in a buffer solution of pH 7 over a period of 10 h. Figure 7 shows the drift characteristics of the planar and SiNW pH sensors under SG and DG operation. SiNW pH sensors show relatively lower drift rates of 1.8 mV h^–1^ in SG mode and 8.3 mV h^–1^ in DG mode than those of the planar pH sensors, at 1.9 mV h^–1^ in SG mode and 8.0 mV h^–1^ in DG mode. To compare the drift rates for each pH sensor in SG and DG mode, the percentage of variation in the drift rate for the respective pH-sensitivity was considered. The results show that the SiNW DG pH sensors have the lowest drift rate for a pH sensitivity of 0.8%, implying that they have better long-term chemical stability than any other pH sensors tested here. The drift rates of the planar and SiNW pH sensors measured in each operation mode are summarized in Table 3.

## 4. Conclusions

In this study, we developed SiNW DG FETs and compared them with conventional planar DG FETs. Compared to the planar DG FETs, SiNW DG FETs showed not only a higher capacitive-coupling ratio but also a lower off-state leakage current under high-temperature stress. Furthermore, when the SiNW DG FETs were applied in pH sensors, they exhibited a highly enhanced sensitivity of 984.1 mV/pH, exceeding the Nernst limit, and a considerably superior stability characterized by a drift rate of less than 1%. For detecting the potential variation on the sensor surface, the FET-based sensors demonstrated here can be used as potential biological/chemical sensors, beyond applications in pH sensors.[35,36] Thus, we expect that the SiNW DG FET sensor proposed here could be developed into a promising label-free sensor for various biological events, such as enzyme−substrate reactions, antigen−antibody bindings, and nucleic acid hybridizations.

## Disclosure statement

No potential conflict of interest was reported by the authors.

## Funding

This research was financially supported by the Basic Science Research Program through the National Research Foundation of Korea (NRF) funded by the Ministry of Education, Science and Technology [number 2013R1A1A2A10011202, 2014R1A2A1A11050768] and by the Technology Innovation Program [10060155] funded By the Ministry of Trade, industry & Energy (MI, Korea) and the KRIBB Initiative Research Program (KRIBB, Korea).

## Supplemental data

The supplemental material for this paper is available online at http://dx.doi.org/10.1080/14686996.2016.1253409

## Supplementary Material

161021_2._STAM_Supplementary_data_CM160725_r2.docx
