# Supplementary material for: Improved sensing characteristics of dual-gate transistor sensor using silicon nanowire arrays defined by nanoimprint lithography
Source: Sci Technol Adv Mater. 2017 Jan 6;18(1):17–25. doi: 10.1080/14686996.2016.1253409 (PMC5256244; doi:10.1080/14686996.2016.1253409)
Supplement: 161021_2._STAM_Supplementary_data_CM160725_r2.docx [file tsta_a_1253409_sm8776.docx]

**Supplementary Data**

**Improved sensing characteristics of dual-gate transistor sensor using silicon nanowire arrays defined by nanoimprint lithography**

Cheol-Min Lim,^a,∥^ In-Kyu Lee,^b,∥^ Ki Joong Lee,^b^ Young Kyoung Oh,^b^ Yong-Beom Shin,*^,b^ and Won-Ju Cho*^,a^

^a^Department of Electronic Materials Engineering, Kwangwoon University, 20 Gwangwoon-ro, Nowon-gu, Seoul 01897, Republic of Korea; ^b^Hazards Monitoring BioNano Research Center, Korea Research Institute of Bioscience & Biotechnology (KRIBB), 125 Gwahak-ro, Yuseong-gu, Daejeon 34141, Republic of Korea

∥These authors have contributed equally to this work.

^*^Corresponding authors:

Won-Ju Cho ([chowj@kw.ac.kr](mailto:chowj@kw.ac.kr))

Yong-Beom Shin ([ybshin@kribb.re.kr](mailto:ybshin@kribb.re.kr))

**
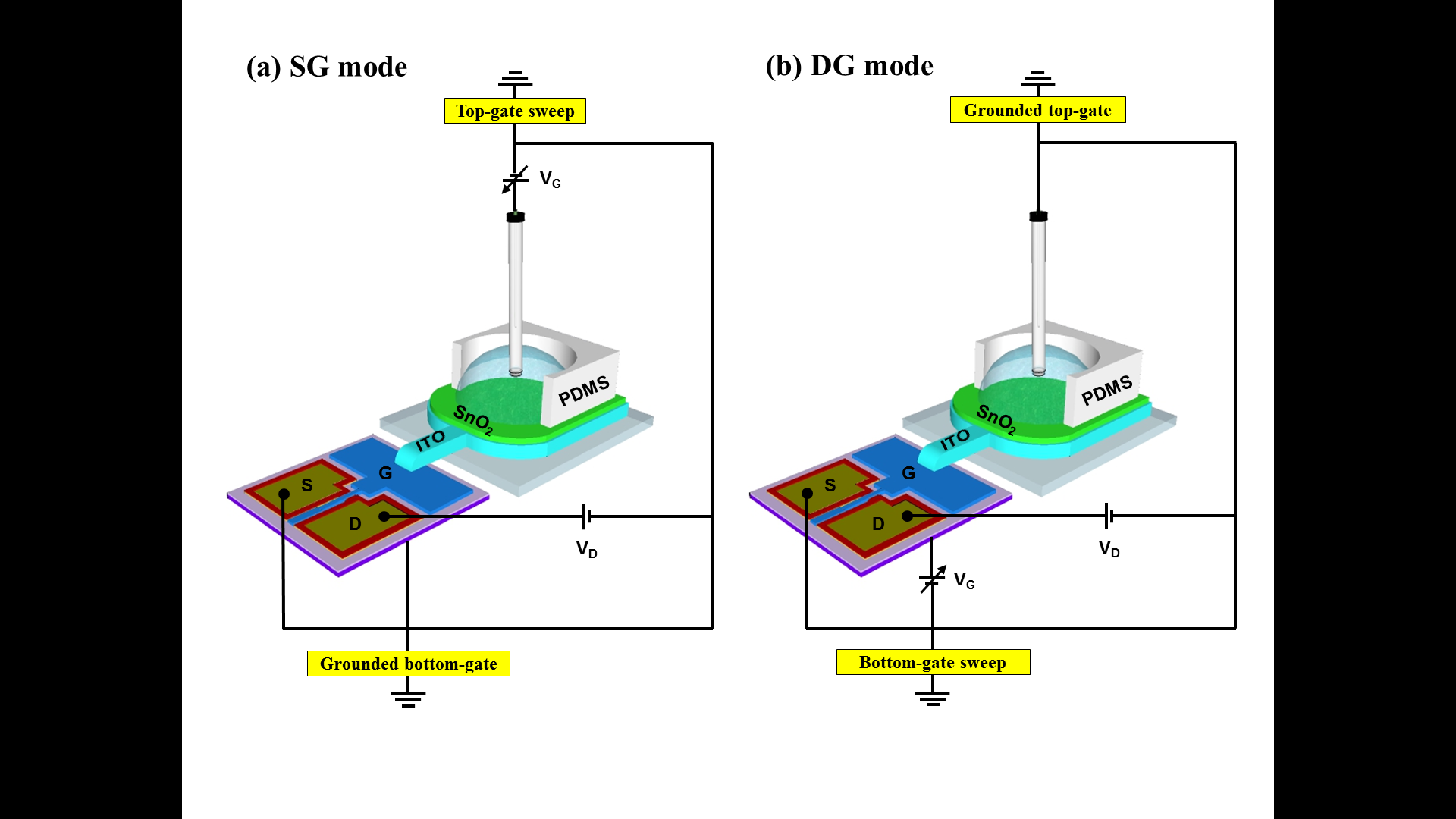
**

Figure S1**.** The description of a) SG operation mode and b) DG operation mode.


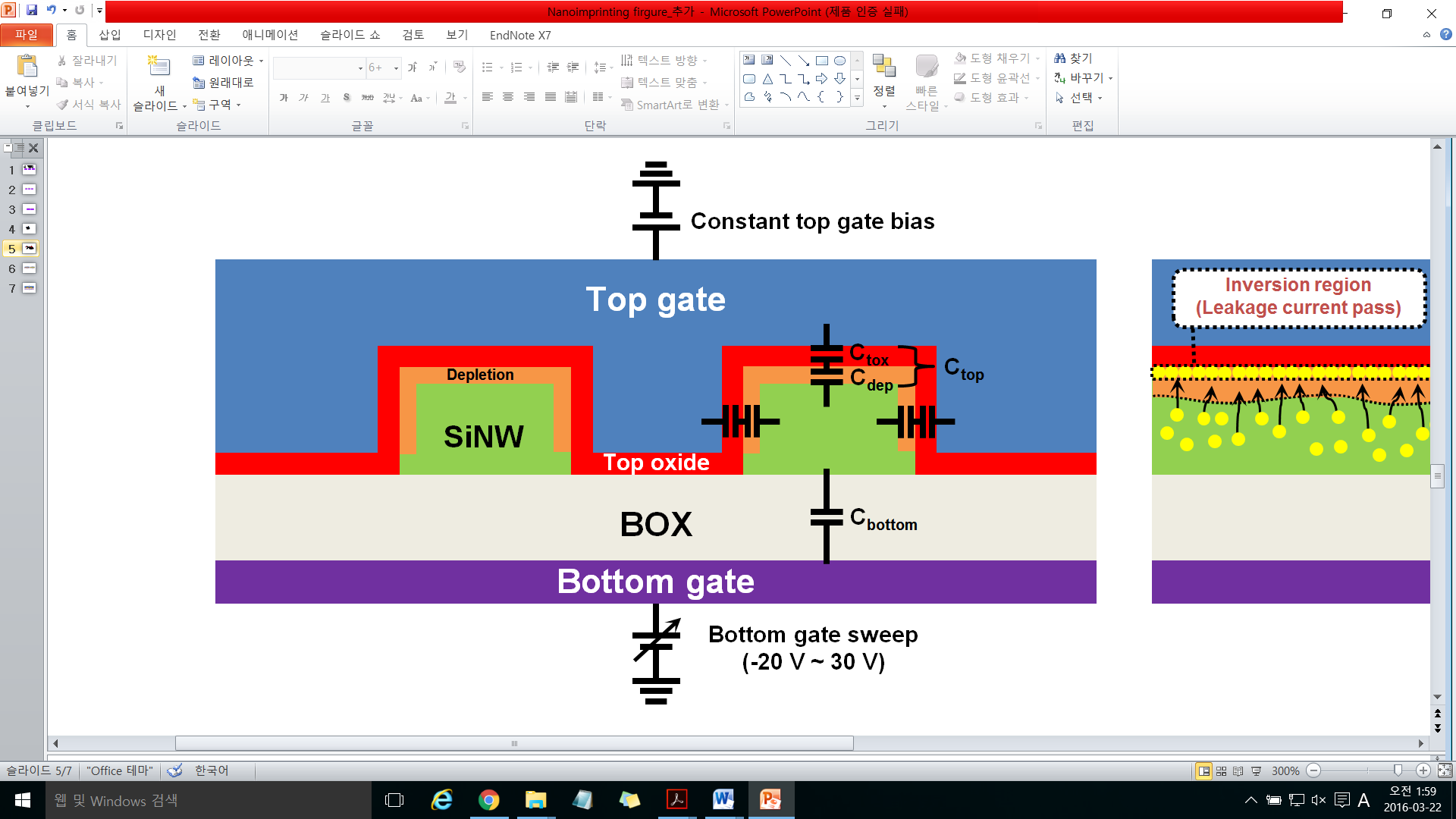


Figure S2**.** Schematic of capacitive-coupling phenomena at the top and bottom interfaces of the SiNW DG FETs.

In principle, capacitive coupling only occurs when the top surface region of the silicon channel is depleted. Thus, the relation between $\Delta V_{_{th}}^{B}$ and $\Delta V_{_{th}}^{T}$ by capacitive coupling can be expressed using Equation (S1) as follows:

$\Delta V_{_{th}}^{B}=\frac{C_{top}}{C_{bottom}}\Delta V_{_{th}}^{T}=\frac{C_{tox}C_{dep}}{C_{bottom}(C_{tox}+C_{dep})}\Delta V_{_{th}}^{T}$, (S1)

where $C_{top}$ and $C_{bottom}$ are the top-gate capacitance per unit area, which is the total capacitance of the top capacitors connected in series, and the bottom-gate capacitance per unit area, respectively. $C_{tox}$ and $C_{dep}$ are the top oxide capacitance per unit area and the depletion capacitance per unit area, respectively.

Meanwhile, capacitance can be described using Equation (S2) as follows:

$C=\varepsilon\frac{A}{d}$, (S2)

where *A* is the area of the plates, *d* is the distance between the two plates, and *ε* is the permittivity of the dielectric medium. In the planar or SiNW DG FETs, $C_{tox}$, $C_{dep}$, and $C_{bottom}$ can only be determined from *A*, as *d* and *ε* are identical in both the devices. Compared to the planar-type silicon DG FETs, the SiNW DG FETs have a larger top surface area and smaller bottom surface area, as shown in Figure S2. In conclusion, this increases the capacitive-coupling ratio of the SiNW DG FETs by leading to an increase in both $C_{tox}$ and $C_{dep}$ and a decrease in $C_{bottom}$.

1. (b)







Figure S3**.** I_D_-Time graphs of (a) planar and (b) SiNW FETs, measured at 25 ^o^C and 120 ^o^C. V_D_, V_TG_, and V_BG_ were set at 50 mV, 600 mV, and -20 V, respectively.

To confirm whether the high-temperature (120 ^o^C) off-state leakage current of the planar DG FET is a result of the inversion layer formed on the top gate, we observed the variation in drain current according to temperature of the planar and SiNW FETs, as shown in Figure S3. Since off-state leakage current of the planar DG FET under high-temperature stress was observed when the bottom gate bias was in the range of -20 V to -10 V, we set the bottom gate bias at -20 V with +600 mV of V_TG_ in this experiment. Consequently, we confirmed that the off-state leakage current of the planar DG FET was approximately 3 nA under the following conditions: 120 ^o^C, V_D_ = 50 mV, V_TG_ = 600 mV, and V_BG_ = -20 V.





Figure S4**.** *I*_D_-*V*_G_ curves of SiNW SG pH sensors for different pH buffer solutions.
